# A Novel Highly Potent Autotaxin/ENPP2 Inhibitor Produces Prolonged Decreases in Plasma Lysophosphatidic Acid Formation *In Vivo* and Regulates Urethral Tension

**DOI:** 10.1371/journal.pone.0093230

**Published:** 2014-04-18

**Authors:** Hiroshi Saga, Akira Ohhata, Akio Hayashi, Makoto Katoh, Tatsuo Maeda, Hirotaka Mizuno, Yuka Takada, Yuka Komichi, Hiroto Ota, Naoya Matsumura, Masami Shibaya, Tetsuya Sugiyama, Shinji Nakade, Katsuya Kishikawa

**Affiliations:** 1 Exploratory Research Laboratories, ONO Pharmaceutical Co., Ltd., Tsukuba, Ibaraki, Japan; 2 Medicinal Chemistry Research Laboratories, ONO Pharmaceutical Co., Ltd., Shimamoto, Mishima, Osaka, Japan; 3 Discovery Technology Laboratories, ONO Pharmaceutical Co., Ltd., Shimamoto, Mishima, Osaka, Japan; 4 Safety Research Laboratories, ONO Phamaceutical Co., Ltd., Sakai, Fukui, Japan; The University of Tennessee Health Science Center, United States of America

## Abstract

Autotaxin, also known as ectonucleotide pyrophosphatase/phosphodiesterase 2 (ENPP2), is a secreted enzyme that has lysophospholipase D activity, which converts lysophosphatidylcholine to bioactive lysophosphatidic acid. Lysophosphatidic acid activates at least six G-protein coupled recpetors, which promote cell proliferation, survival, migration and muscle contraction. These physiological effects become dysfunctional in the pathology of cancer, fibrosis, and pain. To date, several autotaxin/ENPP2 inhibitors have been reported; however, none were able to completely and continuously inhibit autotaxin/ENPP2 *in vivo*. In this study, we report the discovery of a highly potent autotaxin/ENPP2 inhibitor, ONO-8430506, which decreased plasma lysophosphatidic acid formation.

The IC_50_ values of ONO-8540506 for lysophospholipase D activity were 6.4–19 nM for recombinant autotaxin/ENPP2 proteins and 4.7–11.6 nM for plasma from various animal species. Plasma lysophosphatidic acid formation during 1-h incubation was almost completely inhibited by the addition of >300 nM of the compound to human plasma. In addition, when administered orally to rats at a dose of 30 mg/kg, the compound demonstrated good pharmacokinetics in rats and persistently inhibited plasma lysophosphatidic acid formation even at 24 h after administration.

Smooth muscle contraction is a known to be promoted by lysophosphatidic acid. In this study, we showed that dosing rats with ONO-8430506 decreased intraurethral pressure accompanied by urethral relaxation. These findings demonstrate the potential of this autotaxin/ENPP2 inhibitor for the treatment of various diseases caused by lysophosphatidic acid, including urethral obstructive disease such as benign prostatic hyperplasia.

## Introduction

Autotaxin (ATX) was first reported in 1992 [1] as a tumor cell migration factor secreted from melanoma cells. It was elucidated in 1994 that the primary structure of ATX is same as that of ENPP2 [2] – a member of the ectonucleotide pyrophosphatase/phosphodiesterase (ENPP) family. Furthermore, in 2002, plasma LysoPLD activity that produced lysophosphatidic acid (LPA) in circulating blood was found identical to ATX/ENPP2 [3], [4]. ATX/ENPP2 is highly selective for lysophosphatidylcholine (LPC) with an unsaturated fatty-acid side chain [4], and this substrate selectivity was also explained in the analysis of its crystal structure [5], [6].

In 1978, the physiological role of LPA as vasoactive phospholipids was reported [7], and many other biological activities of LPA, such as the promotion of cell proliferation and cell migration, were elucidated in the 1990s. It was first hypothesized that G protein-coupled receptors in plasma membrane were involved in these LPA activities [8]. More recent studies from 1996 onwards revealed that LPA receptors belonging to the EDG and P2Y families mediate the physiological activity of LPA [9]. LPA is implicated in the growth and metastasis of tumor cells [10], chemo-resistance in cancer [11], asthma [12], fibrosis of the lung and kidney [13], [14], neuropathic pain [15], inflammation such as in rheumatism [16] and cardiovascular events [17].

There have been many reports suggesting the involvement of the LPA-producing enzyme ATX/ENPP2 in various diseases. Abnormal vascular formations in the yolk sacs, placentas, and embryos were observed in ATX/ENPP2-knockout mice. These abnormalities led to the death of these animals by embryonic day 10.5 (E10.5) [18]. From these findings, it is considered that ATX/ENPP2 plays an important role in the formation of blood vessels. In addition, symptoms of collagen-induced arthritis were attenuated in mice with conditional ATX/ENPP2 gene deletions, which suggested an involvement of ATX/ENPP2 in chronic inflammation [19]. Similarly, symptoms of nerve injury-induced neuropathic pains are attenuated in mice with heterozygous ATX/ENPP2 gene mutations, suggesting an involvement of ATX/ENPP2 in pain generation [20]. Serum ATX/ENPP2 levels are increased in patients with follicular lymphoma [21] and in patients with intrahepatic cholestasis of pregnancy (ICP) [22], suggesting that the LPA produced by ATX/ENPP2 may affect the pathology of these diseases. Moreover, plasma LPA levels were increased in patients with acute coronary syndrome (ACS), suggesting a possible involvement of ATX/ENPP2 in this disease [17]. Therefore, the development of ATX/ENPP2 inhibitors is expected to provide a novel treatment modality for these diseases.

To date, several compounds, including BrP-LPA [23], S32826 [24], GWJ-A23 [12] [14], HA130 [25], and PF-8380 [26] have been reported as ATX/ENPP2 inhibitors. However, none of these compounds were able to completely and continuously inhibit the activity of ATX/ENPP2 *in vivo*.

In this article, we report the highly potent inhibitory effects of a new ATX/ENPP2 inhibitor, ONO-8430506, which we discovered and developed. The compound showed efficient inhibition of LPA formation, with IC_50_ values of approximately 10 nM with both recombinant and plasma derived ATX/ENPP2 from various animal species. In addition, the compound, when given orally to rats, almost completely inhibited the formation of LPAs in rat plasma even at 24 h after administration. Furthermore, administration of the compound led to decreased intraurethral pressure (IUP) accompanied by urethral relaxation *in vivo*. We conclude from these findings that ONO-8430506 is likely to be effective for the treatment of various diseases caused by excessive LPA signaling.

## Results

### *In vitro* activity of a novel ATX/ENPP2 inhibitor

The IC_50_ values of ONO-8430506 for the LysoPLD activity of recombinant human ATX/ENPP2 were 5.1 nM in an assay using synthetic fluorescent substrate FS-3 and 4.5 nM in an assay using a natural substrate 16:0-LPC. This inhibitory activity is stronger than that of other known ATX/ENPP2 inhibitors such as HA130 and S32826 (Table 1). In addition, an assay using 16:0-LPC demonstrated long-lasting inhibition of ATX/ENPP2; for instance, the IC_50_ of ONO-8430506 was still low at 10.2 nM, even after approximately 15 h of incubation, as opposed to markedly decreased inhibitory activities of HA130 and S32826 (IC_50_ was 580 nM for HA130 and >10,000 nM for S32826 after incubation for about 15 h). Furthermore, ONO-8430506 inhibited both mouse and rat recombinant ATX/ENPP2 with similar IC_50_ values among the three animal species tested (Table 2). We also tested the effects of the inhibitors on the LysoPLD activity using 16:0-LPC as a substrate in human, monkey, dog, rat, and mouse plasma (Table 2) with similar IC_50_ values as found with recombinant ATX/ENPP2.

**Table 1:** *In vitro* Inhibition of Recombinant ATX/ENPP2.

		*In vitro* IC_50_ (nM)	
	Recombinant	human ATX/ENPP2	
	FS-3 (2 h)	Choline (30 min)	Choline (O/N)
ONO-8430506	5.1	4.5	10.2
HA130	120	43	580
S32826	17	66	>10,000

ATX = Autotaxin; ENPP2 = ectonucleotide pyrophosphatase/phosphodiesterase 2.

**Table 2:** *In vitro* Inhibition of Plasma LysoPLD Activity.

*in vitro activity*		IC_50_ (nM)
Recombinant	Human	10.2±3.8
ATX/ENPP2	Rat	19.0±8.1
	Mouse	6.4±0.98
Plasma	Human	5.5±0.32
LysoPLD activity	Monkey	11.6±0.52
	Dog	4.7±0.35
	Rat	5.8±0.62
	Mouse	11.1±2.2

LysoPLD = lysophospholipase D.

IC_50_ values of ONO-8430506 and other inhibitors for recombinant human ATX/ENPP2 when a synthetic fluorescent substrate (FS-3) or a biological substrate (16:0-LPC) was used are shown for (A). IC_50_ values of ONO-8430506 for recombinant ATX/ENPP2 protein enzyme activity and LysoPLD activity in plasma samples from various species when incubated in the presence of a biological substrate 16:0-LPC are shown for (B). When 16:0-LPC was used as a substrate, the enzyme activity was determined based on the amount of choline generated. The IC_50_ values means ± S.D. of three independent experiments.

**Figure 1:**
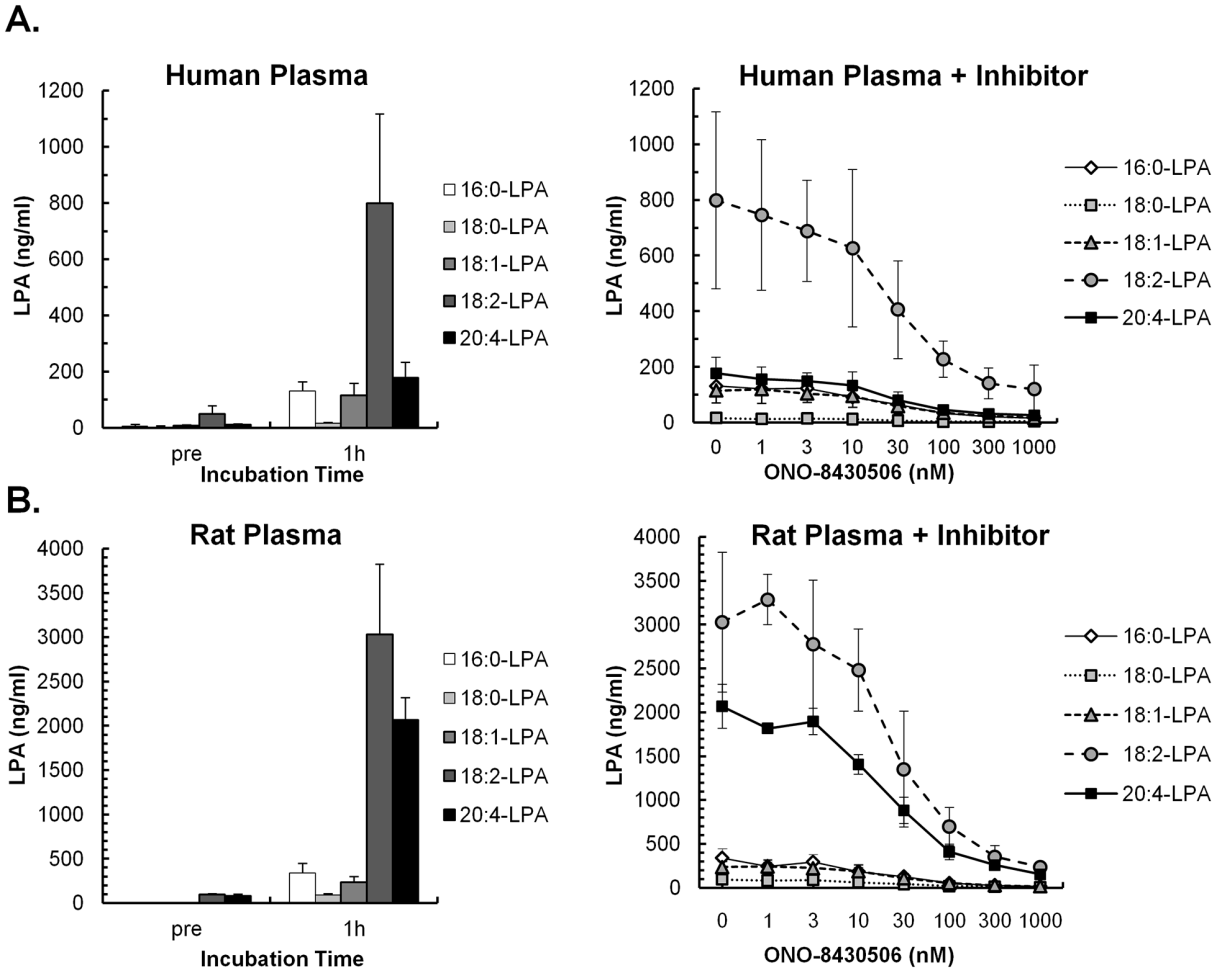
*In vitro* Inhibition of LPA Formation by ONO-8430506 in Human and Rat Plasma. Human plasma (A) and rat plasma (B) were incubated at 37°C for 1 h. Plasma samples before and after incubation were immediately cooled on ice, LPA was extracted and five molecular species of LPA were quantified by LC-MS/MS. The diagrams on the left show the results from incubation of plasma alone, while the diagrams on the right show the inhibition of LPA formation when plasma was incubated with various concentrations of ONO-8430506. Results are means ± S.D. of four separate experiments in human plasma and two or three experiments in rat plasma.

Most of the LPA in plasma is produced by ATX/ENPP2. In the present study, the concentration of plasma LPAs was determined for each fatty-acid side chain. Palmitoyl-LPA (16:0-LPA), stearoyl-LPA (18:0-LPA), oleoyl-LPA (18:1-LPA), linoleoyl-LPA (18:2-LPA), and arachidonoyl-LPA (20:4-LPA) were the five major LPAs in human plasma, amounting to approximately 80 ng/ml in fresh human plasma. However, this total LPA concentration in human plasma increased approximately 10-fold after a 1-h incubation at 37°C (Fig. 1A). The formation of LPAs in human plasma was inhibited by ONO-8430506 in a dose-dependent manner with IC_50_ values of 20–30 nM for all five major LPAs. Similar inhibition of LPA formation by this compound were also observed in rat plasma, although the total LPA level after incubation was 3–4 times higher in rat plasma than in human plasma (Fig. 1B).

ONO-8430506 was proved to be a selective inhibitor of ATX/ENPP2 as the compound showed no inhibition of other two ENPP family enzymes, ENPP4 of which substrate is a nucleic acid and ENPP6 of which substrate is glycerophosphorylcholine, up to 100 µM. Further, the compound showed no inhibition of radioligand-binding to other approximately sixty pharmacological drug targets, such as neurotransmitter receptors, ion channels and transporters, at 10 µM.

### Pharmacokinetics and pharmacodynamics of the inhibitor in rats

**Figure 2:**
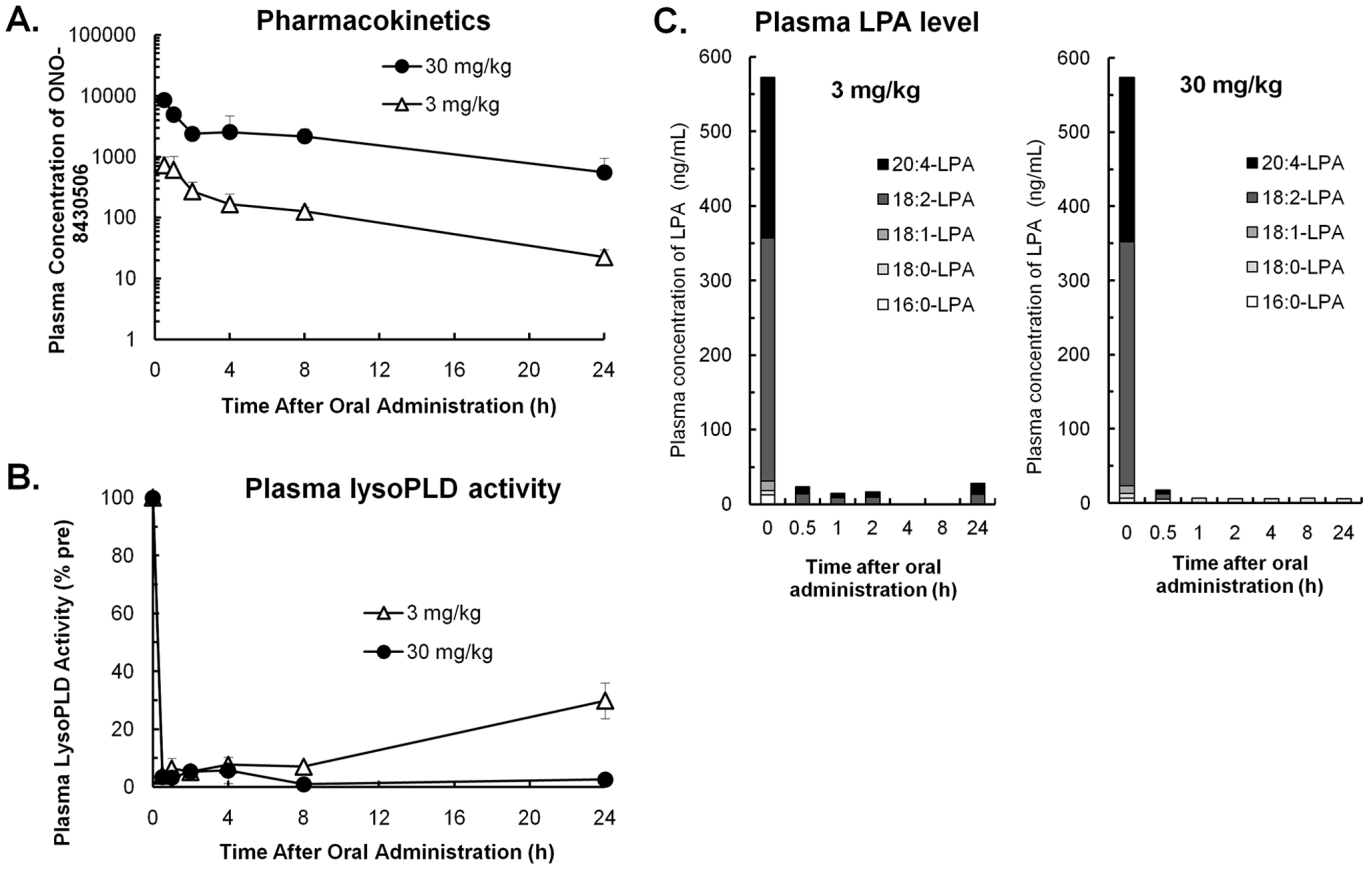
Pharmacokinetics and Pharmacodynamics of the Inhibitor in Rats. Blood was collected at various time points after single oral administration of 3 or 30/kg ONO-8430506 to rats. The time course of changes in plasma concentration of ONO-8430506 (A), plasma *ex vivo* LysoPLD activity (B), and plasma concentrations of various LPAs (C) are shown. Plasma LysoPLD activity is shown relative to LysoPLD activity before administration of the compound. Quantify limit of each LPA from plasma was 5 ng/ml. Results are mean ± S.D. for three rats in each group.

From analyses of the pharmacokinetic profile of ONO-8430506 in rats, the half-life period (T_1/2_) of this compound in circulation blood is about 3 hours (Figure S1, Table S1). The bioavailability which was estimated as the ratio of AUC after oral administration to that after intravenous administration was 52%. In subsequent experiments, we simultaneously examined the time course of changes in plasma ONO-8430506 concentrations, plasma LysoPLD activity, and plasma LPA concentration after single oral administration of the compound (at 3 or 30 mg/kg) to rats. As a result, the concentration of the compound in rat plasma was found to increase in a dose-dependent manner (Fig. 2A). We also examined the time course of changes in LysoPLD activity in rat plasma samples collected at various times after single oral administration of ONO-8430506. LysoPLD activity in rat plasma was inhibited at an efficiency of 90% or higher within 8 h after oral administration of ONO-8430506 (Fig. 2B). At 24 h after the oral administration, 29% of LysoPLD activity in rat plasma had returned in the 3 mg/kg group, whereas the corresponding LysoPLD activity continued to be inhibited in the 30 mg/kg group. Formation of most LPAs was inhibited in rat plasma at 30 min after oral administration of the compound (Fig. 2C). At 24 h after oral administration of ONO-8430506, 4% of 18:2-LPA and 7% of 20:4-LPA returned in the plasma of the 3 mg/kg group, when compared to baseline plasma levels of the corresponding LPAs. There was no significant return of these LPAs in the plasma of the 30 mg/kg group.

As shown above, ONO-8430506 strongly inhibited plasma ATX/ENPP2 activity both *in vitro* and *in vivo*, thereby depleting LPAs from the plasma. In addition, three parameters (plasma compound concentration, plasma LysoPLD activity, and plasma LPA concentration) were found to correlate well with one another. Furthermore, this compound showed no abnormal phenotype after the above stated single administration and no remarkable weight changes during 2 weeks repeated administration in rats.

### Control of urethral tension by LPA and ATX/ENPP2 in rats

**Figure 3:**
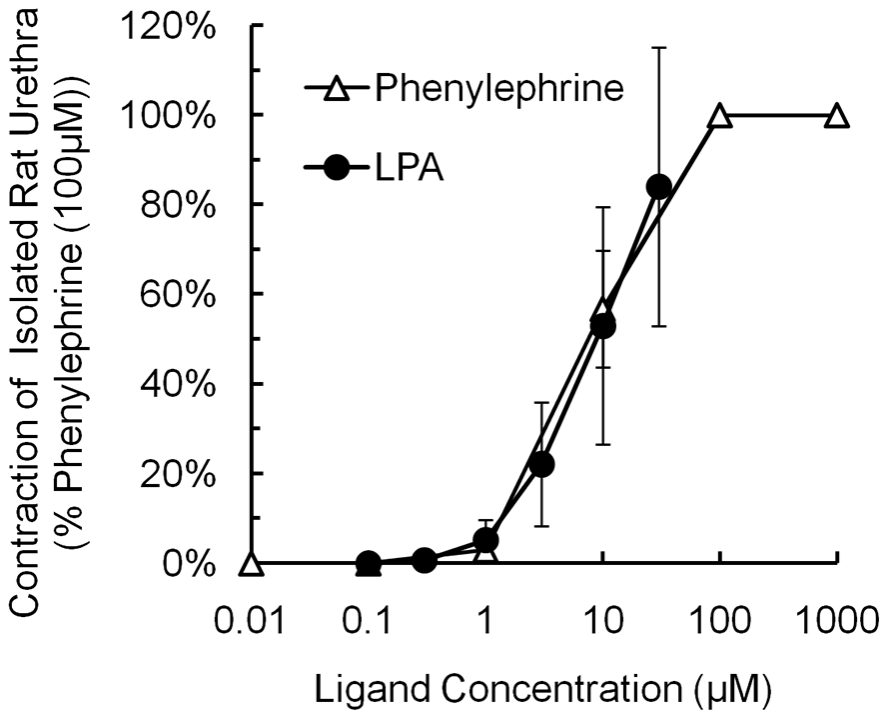
LPA Stimulate the Contraction of Isolated Rat Urethra. The increases in contractile force of isolated rat urethra treated with 18:3-LPA or phenylephrine added to the Magnus apparatus are shown relative to the results for phenylephrine at 100 µM (100%). Results are mean ± S.D. for four urethral specimens.

LPA is a strong smooth muscle-contracting factor, and polyunsaturated LPA exhibits a strong contractile effect on the intestine [27], [28]. We showed that linolenoyl-LPA (18:3-LPA) has a contractile effect in isolated rat urethra, which is comparable to phenylephrine, an α-adrenoceptor agonist (Fig. 3).

**Figure 4:**
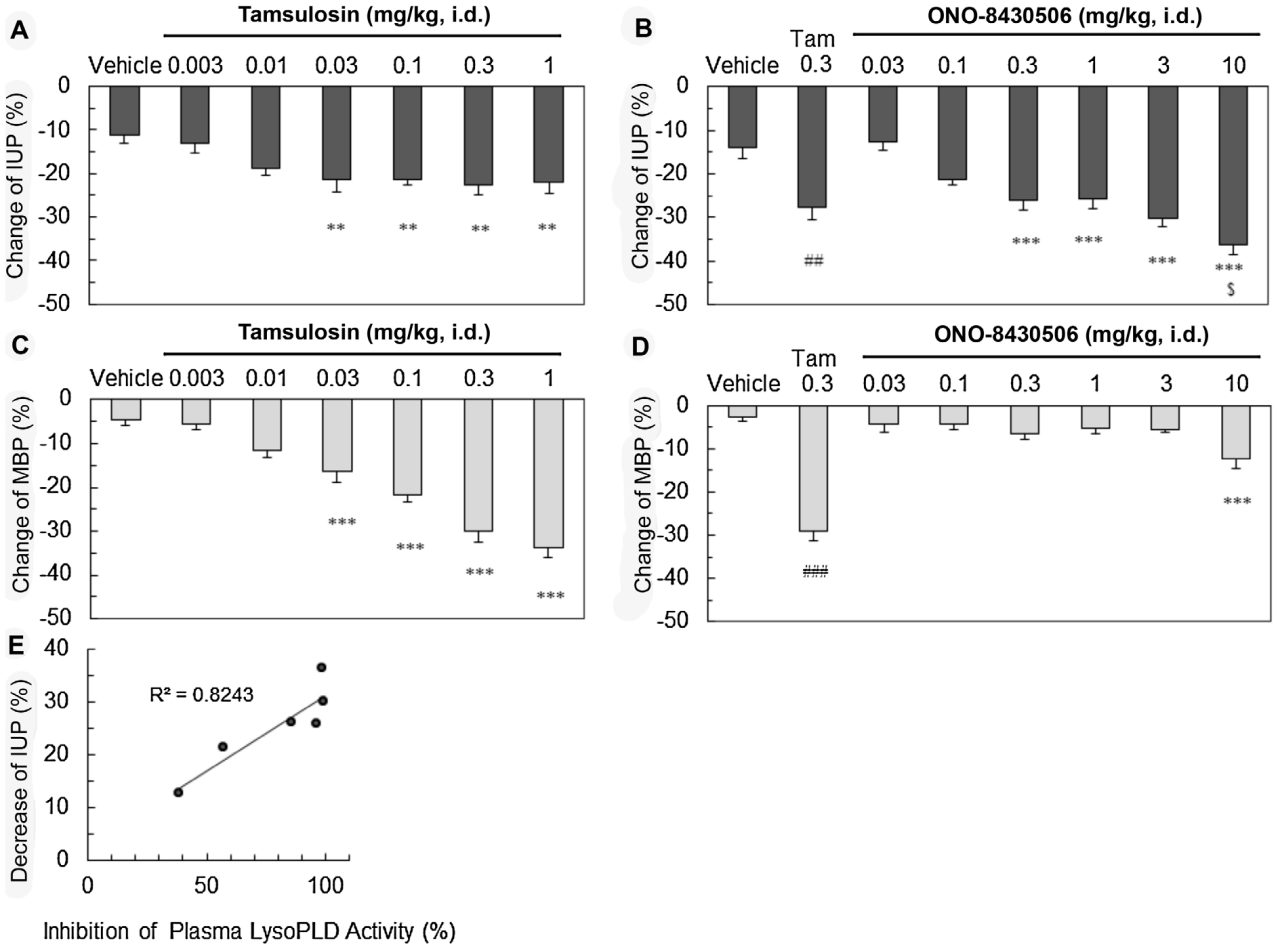
ONO-8430506 regulates *in vivo* Rat Urethral Tension. Male SD rats were anesthetized with urethane and the vehicle, tamsulosin (α_1_-blocker), or ONO-8430506 were administered intraduodenally. The histograms show the maximum percent decreases (%) in IUP (A and B) and maximum percent decreases in mean blood pressure (C and D) during 20-min after intraduodenal administration of each drug. Results are means ± S.E. for nine or ten rats per group. Comparisons between the treatment groups and vehicle control group were performed using the Dunnett's tests or Student's *t* tests. ## p<0.01; ### p<0.001 by Student's *t* test vs Vehicle. ** p<0.01, *** p<0.001 Dunnett test vs Vehicle. $<0.05 by Student's *t* test vs Tam 0.3. A correlation between the relative inhibition (vs. vehicle control) of plasma LysoPLD activity (%) by the compound and the decreases in IUP (%) at 20 min after intraduodenal administration are shown (E).

We hypothesized that inhibition of LPA formation by oral administration of ONO-8430506 should decrease intraurethral pressure (IUP) as an *in vivo* index of urethral tension. The *in vivo* IUP measurement requires rats to be anesthetized by urethane. ONO-8430506 is orally available, however, the orally administrated compound may not be sufficiently absorbed in the intestine due to a low distribution associated with the suppression of the stomach excretion under anesthesia. Therefore, we intraduodenally administrated an α_1-_adrenoceptor antagonist, tamsulosin, in urethane-anesthetized rats according to a previous report [29]. Tamsulosin significantly decreased IUP at doses higher than 0.03 mg/kg, and it showed maximum effect at 0.3 mg/kg (Fig. 4A). Next, we administrated ONO-8430506 in rats, and observed a dose-dependent decrease IUP at doses higher than 0.3 mg/kg. The decreases in IUP by 3 mg/kg ONO-8430506 (30%) was similar to the maximum decrease in IUP by 0.3 mg/kg tamsulosin (28%) whereas that by 10 mg/kg ONO-8430506 (36%) was greater than that by 0.3 mg/kg tamsulosin (p<0.05) (Fig. 4B). Intraduodenally administrated ONO-8430506 up to 3 mg/kg showed no effects on mean blood pressure (MBP) while tamsulosin drastically decreased MBP at the same dose range (Fig. 4C and 4D). The relative inhibition of LysoPLD activity in plasma collected 20 minutes after administration of ONO-8430506 was 39%, 57%, 86%, 97%, 100% and 99% at each dose of 0.03, 0.1, 0.3, 1, 3 and 10 mg/kg. Plasma LysoPLD activity was inhibited >80% by ONO-8430506 in the dose range where it decreased IUP to the same extent as the maximum decrease obtained with tamsulosin (Fig. 4E). These findings suggested that ONO-8430506 may improve urethral obstructive diseases by inhibition of ATX/ENPP2.

## Discussion

We developed a novel, highly potent ATX/ENPP2 inhibitor, a tetrahydrocarboline derivative, called ONO-8430506 as a result of our *in vitro* screening and subsequent development of chemically synthesized ATX/ENPP2 inhibitors [30], [31]. This compound selectively inhibited ATX/ENPP2 with an IC_50_ of approximately 10 nM when tested with the LysoPLD activity of recombinant ATX/ENPP2 and plasma from various animal species (Table 1). This demonstrated that the strong inhibitory activity was not affected by the presence of plasma proteins. While the compound has an IC_50_ of 5.5 nM against plasma LysoPLD assay (Table 2), it had that of 20–30 nM against the generation of various LPA species in plasma (Figure 1). The reaction conditions such as kind of substrate, concentration of substrate, and pH condition were different between two plasma ATX/ENPP2 activity assays, therefore, IC_50_ of ONO-8430506 in plasma LysoPLD assay which reacted under the optimal alkaline pH for the enzyme might be a few-fold more potent than that in plasma LPA formation assay.

In addition, experiments in rats showed that oral administration of 30 mg/kg ONO-8430506 completely inhibited LPA formation in plasma even at 24 h. To date, several compounds including BrP-LPA [23], S32826 [24], and HA130 [25] have been reported as ATX/ENPP2 inhibitors; however, none were able to completely and continuously inhibit ATX/ENPP2 *in vivo*. Another known ATX/ENPP2 inhibitor, PF-8380 [26], also has an inhibitory effect on plasma LPA formation for several hours after oral administration, but the plasma LPA level reportedly returned to the normal baseline level at 24 h after administration. In contrast, the present pharmacokinetic study in rats shows the long-lasting effect of ONO-8430506 in plasma, which is necessary for the inhibition of plasma LysoPLD activity (Fig. 2). We conclude that ONO-8430506 has superior and persistent inhibitory effects on ATX/ENPP2 *in vivo* compared to other inhibitors reported so far.

LPA has a glycerol backbone wherein either the *sn*-1 position or the *sn*-2 position has a fatty-acid side chain attached. The abilities of LPAs to activate receptor molecules depends on the length of fatty acid side chain and the number of unsaturated bonds [32], [33]. ATX/ENPP2 is highly selective for LPCs with an unsaturated fatty-acid chains [4], [5], [6]. We showed that incubation of human or rat plasma at 37°C enhanced the formation of LPAs with an unsaturated fatty-acid side chain in plasma, such as 18:2-LPA and 20:4-LPA. The addition of ONO-8430506 suppressed the formation of these LPA (Fig. 1). In addition, the administration of ONO-8430506 to rats almost completely inhibited the formation of two major LPAs, 18:2-LPA and 20:4-LPA in the plasma (Fig. 2C). Therefore, it was expected that ONO-8430506 would suppress the physiological activity of LPAs with an unsaturated fatty-acid side chain and improve the disease status associated with this activity.

On the other hand, enzyme kinetic analysis suggests a competitive inhibition of ATX/ENPP2 as the mechanism underlying the inhibition of ATX/ENPP2 by ONO-8430506 (Figure S2). In addition, previous crystallographic analyses of ATX/ENPP2 [5], [6] revealed that the substrate, LPC, binds to the hydrophobic pocket of the enzyme. In our X-ray crystallographic analyses, it was confirmed that a structural analogue of ONO-8430506 binds to the same hydrophobic pocket of rat ATX/ENPP2 (results not shown). From these findings, the compound seems to be an inhibitor that competes with LPC for the substrate-binding site of ATX/ENPP2.

The earliest report on physiological effects of LPAs was the study by Tokumura et al [27], [28] concerning contractile effects of LPAs on smooth muscles of isolated intestinal tracts. LPA had a strong smooth muscle contracting effect on the ileum from guinea-pigs and the large intestine from rats. LPAs with a higher degree of unsaturated fatty-acid side chain have a stronger contractile effect, and 18:3-LPA is the most potent. We examined the effects of LPAs on isolated rat urethra and confirmed that 18:3-LPA has a contractile effect on rat urethra (Fig. 3). In addition, an LPA_1_ receptor antagonist inhibited the contractile effect of LPA on rat urethra (results not shown) [34].

In the present study, we determined the effects of inhibiting LPA formation with ONO-8430506 using IUP as an *in vivo* index of urethral tension. The compound was given intraduodenally to rats it decreased IUP in a dose-dependent manner. The maximum decreases in IUP by ONO-8430506 were larger than that by α_1_-blocker, tamsulosin (P<0.05). When compared to tamsulosin, the effects of ONO-8430506 on IUP and blood pressure appeared to be highly selective for the urethra. In another experiment in conscious rats, the compound had no influence on heart rate by oral administration up to 30 mg/kg (data not shown). Thus, ONO-8430506 seems to have a smaller impact on the cardiovascular system.

The relative inhibition of plasma LysoPLD activity by ONO-8430506 was 80 to 90% at the dose range where ONO-8430506 decreased IUP to a similar extent to the maximum decreases observed with tamsulosin. Such a high inhibition rate of plasma LysoPLD activity can be sustained throughout 24 h by single oral administration of ONO-8430506 at 30 mg/kg (Fig. 2). Furthermore, it was reported that expressions of ATX/ENPP2 and certain types of LPA receptors are increased in the prostate gland of patients with prostatic hyperplasia [35]. Therefore, ONO-8430506 has a favorable profile as a potent drug for decreasing IUP in the treatment of urinary disturbances, such as benign prostatic hyperplasia, for which tamsulosin is presently prescribed.

Recently, involvement of ATX/LPA for immune inflammatory response has been reported for asthma [12], pulmonary fibrosis [13] [14] and lymphocyte homing to lymphoid organs [36]. LPA produced by ATX/ENPP2 is reported to work in the lymphocyte trafficking process in the lymph node. On the other hand, we confirmed that this compound shows no remarkable changes in immune associated organs after 2 weeks repeated dosing in rats. Although it is difficult to discuss the cause of the difference at this time, there may be some difference between normal and pathological conditions.

These findings suggest that a novel ATX/ENPP2 inhibitor, ONO-8430506, may have a favorable effect on urethral obstructive diseases, in addition to other diseases, such as cancer, fibrosis, and rheumatism, where the causal relationship of ATX/ENPP2 has already been established.

## Materials and Methods

### Reagents

1-palmitoyl-2-hydroxy-*sn*-glycero-3-phosphate (16:0-LPA), 1-stearoyl-2-hydroxy-*sn*-glycero-3-phosphate (18:0-LPA), 1-oleoyl-2-hydroxy-*sn*-glycero-3-phosphate (18:1-LPA), 1-palmitoyl-2-hydroxy-*sn*-glycero-3-phosphocholine (16:0-LPC) and 1-myristoyl-2-hydroxy-*sn*-glycero-3-phosphocholine (14:0-LPC) were purchased from Avanti Polar Lipids, Inc. (Alabaster, AL). D-(+)-*sn*-1-O-linolenoyl-glyceryl-3-phosphate (18:2-LPA), D-(+)-*sn*-1-O-arachidonoyl-glyceryl-3-phosphate (20:4-LPA),FS-3, was purchased from Echelon Biosciences Inc. (Salt Lake City, UT). Amplex Red was purchased from Life Technologies Corporation (Carlsbad, CA). HA130, was purchased from Tocris Biosciences (Bristol, UK). Bovine serum albumin (BSA, essentially fatty acid-free), 4-aminoantipyrine, horseradish peroxidase, and S32826, were purchased from Sigma-Aldrich Japan K. K. (Tokyo, Japan). TOOS was purchased from Dojindo Laboratories (Kumamoto, Japan). ONO-8430506 and tamsulosin hydrochloride were synthesized by Ono Pharmaceutical Co., Ltd.

Pooled human plasma, (Sodium Heparin) was purchased from Bioresource Technology Inc. (Miramar, FL). Fresh human plasma was obtained from healthy volunteers, and plasma samples were obtained from 14-week-old female BALB/c mice (Charles River Laboratories Japan, Inc.), 7-week-old male SD (IGS) rats (Charles River Laboratories Japan, Inc.), and 12 to 35-month-old male TOYO beagles (Kitayama LABES Co., Ltd., Japan). Blood was collected from each animal species using a heparinized syringe, centrifuged at 4°C and 8,000 *g* for 5 min, and plasma samples obtained. Macaque monkey plasma was purchased from Hamri Co., Ltd. (Ibaraki, Japan). These plasma samples were stored at −80°C.

#### Research ethics

All animal experiments were conducted in accordance with the internal regulations for care and use of laboratory animals by Ono Pharmaceutical Co., Ltd., and under the approval of the Ethics Committee for Animal Experiments of Ono Pharmaceutical Co., Ltd. Maximum efforts were made to minimize suffering, and all surgery and measurement under inserting catheter ware performed under deep anesthesia. Fresh blood was collected from healthy volunteers in accordance with ‘Ethical Guideline for Researchers Involved in Handling Human Tissues’ by Ono Pharmaceutical Co., Ltd., and under the approval of the Ethics Committee for Experiments using Human Tissues of Ono Pharmaceutical Co., Ltd. We got the informed consent from all healthy volunteers in a document.

### Recombinant ATX/ENPP2

cDNA of human ATX/ENPP2-(His)_6_ was cloned using 5′-TACCGAGCTCGGATCGCCACCATGACTTCGAAATTTCTCTTG-3′ and 5′-TAGACTCGAGCGGCCTCACTTGTCGTCATCGTCTTTGTAGTCGGCTTGTAATAATGGTTGAGC-3′ primers using the ‘brain’ and ‘liver’ tissues in the human multiple tissue cDNA panel-1 from BD Biosciences. Human ATX/ENPP2-(His)_6_ cDNA was subcloned into the transfer vector pPSC12, and then the resulting expression vector pPSC12/hENPP2 and baculovirus AcNPV were co-transfected into Sf9 cells. Viral particles from the culture supernatant were purified and amplified, and, finally, a solution of the viruses carrying recombinant human ATX/ENPP2 gene (hENPP2/AcNPV) was obtained.

cDNA of rat ATX/ENPP2 was synthesized by attaching a His-tag to the *C*-terminus of the open reading frame (ORF) region corresponding to the sequence registered in the GenBank database (ID: NM057104). Rat ATX/ENPP2 cDNA was subcloned into the transfer vector pSC8, and then the resulting expression vector pPSC8/rENPP2 and baculovirus AcNPV were co-transfected into Sf9 cells. Viral particles from the culture supernatant were purified and amplified and, finally, a solution of the viruses carrying recombinant rat ATX/ENPP2 gene (rENPP2/AcNPV) was obtained.

Solutions of viruses carrying the recombinant mouse ATX/ENPP2 gene (mENPP2/AcNPV) were supplied by Professor Junken Aoki of Tohoku University. The solution of viruses carrying the gene of each recombinant ATX/ENPP2 protein (hENPP2/AcNPV, rENPP2/AcNPV, or mENPP2/AcNPV) was infected into expresSF+ cells (Nosan Corporation, Japan). The resulting culture supernatant was collected and the recombinant ATX/ENPP2 protein for each animal species was purified using a His-Tag affinity column, After purification, each ATX/ENPP2 protein was dissolved in PBS containing 20% glycerol and stored at −80°C.

### *In vitro* LysoPLD activity assay

#### FS-3 assay

Recombinant ATX/ENPP2 protein (2 µg/ml) and various concentrations of the inhibitors were mixed and incubated at 37°C for 2 h in 100 mM Tris-HCl buffer (pH 8.0) containing 1%DMSO, 500 mM NaCl, 5 mM MgCl_2_, and 0.05% Triton X-100, in the presence of a fluorescent substrate FS-3 (1 µM). After incubation, fluorescence at 520 nm with excitation at 494 nm was measured.

#### Choline release assay

In the choline release assay, a series of reaction mixtures containing different concentrations of the inhibitors were tested in parallel. Recombinant ATX/ENPP2 was dissolved in 100 mM Tris-HCl buffer (pH 9.0) containing 500 mM NaCl, 5 mM MgCl_2_ and 0.05% Triton X-100. First, the aqueous solution of recombinant ATX/ENPP2 (final concentration 2 µg/ml) was added to the reaction mixture containing different concentrations of the inhibitors, 1 mM 16:0-LPC, and DMSO 1%. Then, each mixture was incubated at 37°C for 30 min (primary reaction). A samples of each reaction mixture was subjected to the secondary incubation at 37°C for 30 min with 10 times the volume of 100 µM Amplex Red in 50 mM Tris-HCl buffer (pH 8.0), 1 U/ml horseradish peroxidase, 150 mU/ml choline oxidase and 5 mM CaCl_2_,. Fluorescence at 590 nm was measured with excitation at 544 nm and the amount of choline released in each reaction mixture was quantified based on a calibration curve created from a choline chloride standard.

A series of reaction mixtures containing different concentrations of the inhibitors were also tested in parallel using an overnight incubation. First, the aqueous solution of recombinant ATX/ENPP2 (final concentration 2 µg/ml) or plasma samples from various animal species (final concentration 40%) was added to the reaction mixture containing different concentrations of ATX/ENPP2 inhibitors, 1 mM 16:0-LPC, and 1% DMSO. Then, each mixture was incubated at 37°C for 15 h (primary reaction). Samples of each mixture were subjected to the secondary reaction with 10 volumes of 300 µM TOOS in 100 mM Tris-HCl solution (pH 8.5), 500 µM 4-aminoantipyrine, 600 mU/ml horseradish peroxidase, 3 U/ml choline oxidase, and 5 mM CaCl_2_, at 37°C for 20 min (secondary reaction). The amount of choline released in each reaction mixture was quantified from the absorbance at 555 nm using a blank obtained from incubations, which did not contain ATX/ENPP2 or plasma, using calibration curve created with the choline chloride standard.

#### Calculation of Inhibitory activity

Measurement points of choline production were plotted against the concentration of the inhibitor and the theoretical curve to obtain the IC_50_ value of the inhibitor for LysoPLD activity was obtained using the Activity Base version 7.1.0.39 software from ID Business Solutions Ltd.

#### *In vitro* LPA formation assay

The inhibitor (5 µl) was added to human or rat plasma (95 µl) incubated on ice. The reaction was subsequently incubated for 1-h at 37°C, after which the reaction mixture was placed on ice and LPA was extracted immediately. The concentrations of molecular species of LPA before and after the incubation were determined using an internal reference standard added to each same plasma sample and by comparison to a calibration curve for each LPA. The detailed procedures were as follows: 16:0-LPA, 18:0-LPA, 18:1-LPA, 18:2-LPA, and 20:4-LPA were dissolved together in a chloroform/acetic acid (4∶1) at a concentration of 1,000 µg/ml. This solution was further diluted to 0.5 to 100,000 ng/ml with a chloroform/methanol/ethanol/acetic acid (2∶1∶1∶1). The dilution series thus obtained (*plus* the solvent control as the ‘0 ng/ml’ point) was used for creating a calibration curve for LPAs. A 100-µl portion from each point of the dilution series was mixed with 100 µl of 0.2% fatty acid free BSA-containing physiological saline, and the mixture (200 µl) was further diluted with 2,000 µl of chloroform/methanol/ethanol (20∶40∶40) containing the internal reference molecule (200 ng/ml). In parallel, 100 µl of the human plasma test sample either before or after the 1-h incubation at 37°C was mixed with 100 µl of chloroform/methanol/ethanol/acetic acid (2∶1∶1∶1) and 200 µl of the mixture was further diluted with 2,000 µl of chloroform/methanol/ethanol (20∶40∶40) containing the internal reference molecule (200 ng/ml). Mixtures were then centrifuged at 4°C and 2,500 rpm for 10 min and the upper organic layer was evaporated to dryness under reduced pressure and was analyzed by LC-MS/MS to determine the concentration of each LPA.

LC-MS/MS was performed using Alliance 2795 HPLC system and Quattro Ultima API triple quadupole mass spectrometer (Waters Co., Ltd.), equipped with electrospray ionisation (ESI) source. HPLC separation were achieved using Unison UK-C18 reverse-phase LC column (3 µm particle size, 30 mm×2 mm i.d.) at 50°C, sample injection volume was 5 µl at a flow rate of 0.2 ml/min. The mobile phase included A: ultra pure water with 6.5 mM NH_4_HCO_3_ and 1 mM NaH_2_PO_4_, and B: acetonitrile/methanol (4∶1), purge solvent included mobile phase A/B (9∶1). The LC gradient started from 100% A to 100% B in 2 min, kept at 100% B for 10 min and finally back to initial phase. Every LPA species were monitored MRM (Multiple Reaction Monitoring) negative-ion mode, selected [M-H]- as parent negative-ion, m/z:153.0 as daughter negative-ion. Conditions of MRM analysis included a capillary voltage of 3 kV, a cone nitrogen gas flow of 46 L/h, a source temperature of 120°C, a desolvation temperature of 350°C, a cone voltage/collision energy of 80 V/29 eV for 16:0-LPA, 18:0-LPA, 18:1-LPA, 53 V/23 eV for 18:2-LPA, and 44 V/23 eV for 20:4-LPA with argon gas in collision cell.

### Pharmacokinetics (PK) and pharmacodynamics (PD) in rats

#### Administration and blood sampling in rats for the simultaneous measurement of PK/PD

A solution of ONO-8430506 was prepared by dissolving the compound in Wellsolve/water (1∶3) mixed solvent containing 1.1 times the molar equivalent of NaOH. This solution was given orally to 8-week-old male Crl∶CD(SD) rats using an oral route. Heparinized blood was collected from the jugular vein under awake but constrained conditions at 30 min before drug administration, at various times after administration. Blood samples were immediately cooled on ice and then centrifuged at 8,000 *g* and 4°C for 5 min. Plasma samples were stored at −80°C and the plasma concentration of the compound, LPAs, and *ex vivo* LysoPLD activity were determined.

#### Measurement of the compound concentration in plasma

Twenty-volumes of acetonitrile/ethanol (7∶3) containing the internal reference was added to each plasma sample. The whole mixture was transferred into a 96-well filter plate (Multiscreen Solvinert, 0.45 µm pore-size, low-binding hydrophilic PTFE, Millipore Corp.), which was then centrifuged at 4°C and 1,600 rpm for 5 min. The same volume of distilled water was added to the filtrate in each well, and the plasma (containing the internal reference) remaining in each well was assigned to different test conditions in an assay. The concentration of each analyte in each plasma sample was calculated from its relative peak area (*vs.* the internal reference) in the LC-MS/MS chromatogram.

#### *Ex vivo* plasma LysoPLD activity

Each plasma sample was diluted 10-fold with 500 mM NaCl in 100 mM Tris-HCl buffer (pH 9.0), 5 mM MgCl_2_, and 0.05% Triton X-100. To the plasma solution, 14:0-LPC was added (final concentration of 3 mM) as the substrate to determine the *ex vivo* LysoPLD activity in plasma. The test plasma samples were incubated at 37°C for 6 h (primary reaction). Second reaction is the same as *in vitro* LysoPLD activity assay by choline release method described above.

### Contraction of isolated urethral muscle

#### Preparation of LPA

Linolenoyl-lysophosphatidylcholine (18:3-LPC) was produced from 1,2-dilinolenoyl-*sn*-glycerol-3-phosphocholine (18:3-PC) (Avanti Corp.) using phospholipase A_2_ (PLA_2_, from bee venom, Sigma). To the solution of 18:3-PC (50 mg/ml) in diethyl ether/methanol (85∶15), PLA_2_ was added at 2.4 U/mg 18:3-PC. The reaction mixture was incubated at room temperature overnight while stirring and the solvent was removed by evaporation. The remaining 18:3-LPC was then extracted into the ether phase by washing five times with a diethyl ether/methanol (95∶5). Next, linolenoyl-lysophosphatidic acid (18:3-LPA) was produced from 18:3-LPC using phospholipase D (PLD) from *Streptomyces chromofuscus* (Sigma-Aldrich Japan). PLD (final concentration of 333 U/ml) was added to the solution of 10 mg/ml of 18:3-LPC in 200 mM Tris-HCl buffer (pH 7.4) containing 5 mM NaF, The reaction mixture was incubated at room temperature overnight while stirring. The reaction mixture was then titrated to pH 2–3 with HCl. The 18:3-LPA remaining in the aqueous solution was then extracted into chloroform phase by washing with chloroform several times. The organic layer containing 18:3-LPA was neutralized with 28% ammonia water. The solvent was concentrated and dissolved in a chloroform/methanol (70∶30). This solution was then applied onto a silica gel column (Wakogel C-200, Wako Pure Chemical Industries, Ltd., Japan). Three solvents were used to eluent 18:3-LPA in the following order: chloroform/methanol (70∶30); chloroform/methanol/distilled water (65∶35∶5) and finally chloroform/methanol/distilled water (50∶50∶5), and eluted fractions containing LPA were detect by thin layer chromatography. The purified 18:3-LPA was stored at −20°C under nitrogen gas. The 18:3-LPA solution was prepared at a concentration of 1 mM in Dulbecco's PBS (Invitrogen) containing 0.25% BSA, and was stored at −80°C before use.

#### Measurement of the tension generated in isolated urethra

Krebs-Henseleit buffer (112 mM NaCl, 5.9 mM KCl, 2.0 mM CaCl_2_, 1.2 mM MgCl_2_, 1.2 mM NaH_2_PO_4_, 25.0 mM NaHCO_3_ and 11.5 mM glucose) was prepared and aerated with 95%O_2_+5%CO_2_. 8-week old male CD (SD) IGS rats (Charles River Laboratories Japan, Inc.) were decapitated and exsanguinated. The urethra was excised and immediately immersed in ice-cooled Krebs-Henseleit buffer. The excised urethra was cut open and flattened using ophthalmologic scissors. The opened and flattened urethra was cut along the circular muscle into ‘ring-shaped’ strips 2–3 mm in width×3–4 mm in length using a razor. One ‘ring-shaped’ urethral specimen was prepared for each male rat.

Each prepared urethral specimen was hung inside a 5-ml Magnus tube filled with Krebs-Henseleit buffer (30°C). A resting tension of approximately 0.5 g was applied to the specimen for 60 min until it was stabilized. Thereafter, contraction motions of the specimen were recorded on a linear recorder (Model WR3320, Graphtec Corp.) after being amplified by a torsion-pressure amplifier (Models AP-641G and AP-601G, Nihon Kohden Corporation) and a force displacement pick-up transducer (Model TB-611T, Nihon Kohden Corporation). After being hung inside a Magnus tube, the urethral specimen was washed with Krebs-Henseleit buffer on an as needed basis. If necessary, the medium was diluted with physiological saline (Otsuka Pharmaceutical Co., Ltd.). Phenylephrine (10 nM to 1 mM, Sigma-Aldrich) or 18:3-LPA (100 nM to 30 µM) was cumulatively added to the medium and the contractile force of each urethral specimen was measured.

### Measurement of intraurethral pressure (IUP) in rats

Male Crl∶CD(SD) rats (7–8 weeks old) were anesthetized by intraperitoneal administration of urethane at a dose of 1.5 g/5 ml/kg. An arterial catheter (JMS cut-down tube; C3, JMS, Japan) was inserted into the common carotid artery to monitor blood pressure. After the abdomen was opened, another catheter (inner diameter, 0.9 mm; outer diameter, 1.5 mm; Shin-Etsu Polymer, Japan) was inserted into the duodenum and held in place with a suture thread. The abdomen was then cut along the median line and the urethra was ligated in the proximity of the pubic bone. For measurement of intraurethral pressure (IUP), a urethral catheter (JMS cut-down tube; C3, JMS Inc., Japan) was inserted from the top side of the bladder through the urethra and held in place by ligation at the bladder neck [29]. Finally, both the arterial catheter and urethral catheter were connected to a pressure transducer (Nihon Kohden, Japan), and the rat was placed on a warm mat (37°C). Blood pressure and IUP were measured by a recorder (Graphtec, Japan) using a torsion-pressure amplifier (Nihon Kohden, Japan).

ONO-8430506 was dissolved in an aqueous solution containing 1.1 times the equivalent amount of NaOH. After confirming stabilization of IUP, rats received either the inhibitor or tamsulosin at a dose of 2 ml/kg in an aqueous solution through the duodenal catheter. IUP and blood pressure were measured continuously during the 20-min after administration of either the inhibitor or tamsulosin. At the end of the 20-min period, heparinized blood was collected from the jugular vein of the rat to prepare a plasma sample. Rats were then euthanized by intravenous administration of 1 ml of pentobarbital (Somunopentyl, Kyoritsu Seiyaku Corporation). Postmortem IUP was measured until the lowest IUP value was reached and stabilized. The maximum percent decreases relative to the difference between baseline IUP and postmortem IUP was calculated to measure the change from baseline in IUP during the 20-min after drug administration.

## Supporting Information

Figure S1 Pharmacokinetics in Rats. After intravenous (at 0.3 mg/kg) or oral (at 1 mg/kg) administration of a single dose of ONO-8430506 to rats under fasting conditions, blood was collected at various time points. The time course of changes in plasma concentration of unchanged compound is shown. The data are given as the mean ± S.D. for three rats in each dose group.
(TIF)

Figure S2 Enzyme Kinetics Analysis. (A) Inhibitory curves of recombinant human ATX/ENPP2 by ONO-8430506 for each concentrations of fluorescent substrate FS-3 (•: 200 nM, ▪: 500 nM, ▴: 1 µM, ▾: 2 µM, ♦: 5 µM). (B) Lineweaver-Burk plot analysis of ATX/ENPP2 inhibition by ONO-8430506 (•: no inhibitor, ▪: 100 pM, ▴: 300 pM, ▾: 1 nM, ♦: 3 nM, ○: 10 nM, □: 30 nM, ▵: 100 nM, ▿: 300 nM). (C) Hanes-Woolf plot analysis of ATX/ENPP2 inhibition by ONO-8430506 (•: no inhibitor, ▪: 100 pM, ▴: 300 pM, ▾: 1 nM, ♦: 3 nM, ○: 10 nM, □: 30 nM, ▵: 100 nM, ▿: 300 nM). ATX/ENPP2 inhibition type by ONO-8430506 is speculated as a competitive type, since Lineweaver-Burk plot crosses at a single point on Y-axis and Hanes-Woolf plot represents parallel lines.
(TIF)

File S1 Contains the following: Method S1 and Method S2.
(DOC)

Table S1 Pharmacokinetics Parameter in Rats. From the time course of changes in plasma concentration of ONO-8430506 (shown in Figure S1), the pharmacokinetics parameters of the compound in rats were determined.
(TIF)
