# Supplementary figures and images for: A Novel Highly Potent Autotaxin/ENPP2 Inhibitor Produces Prolonged Decreases in Plasma Lysophosphatidic Acid Formation In Vivo and Regulates Urethral Tension
Source: PLoS One. 2014 Apr 18;9(4):e93230. doi: 10.1371/journal.pone.0093230 (PMC3991570; doi:10.1371/journal.pone.0093230)

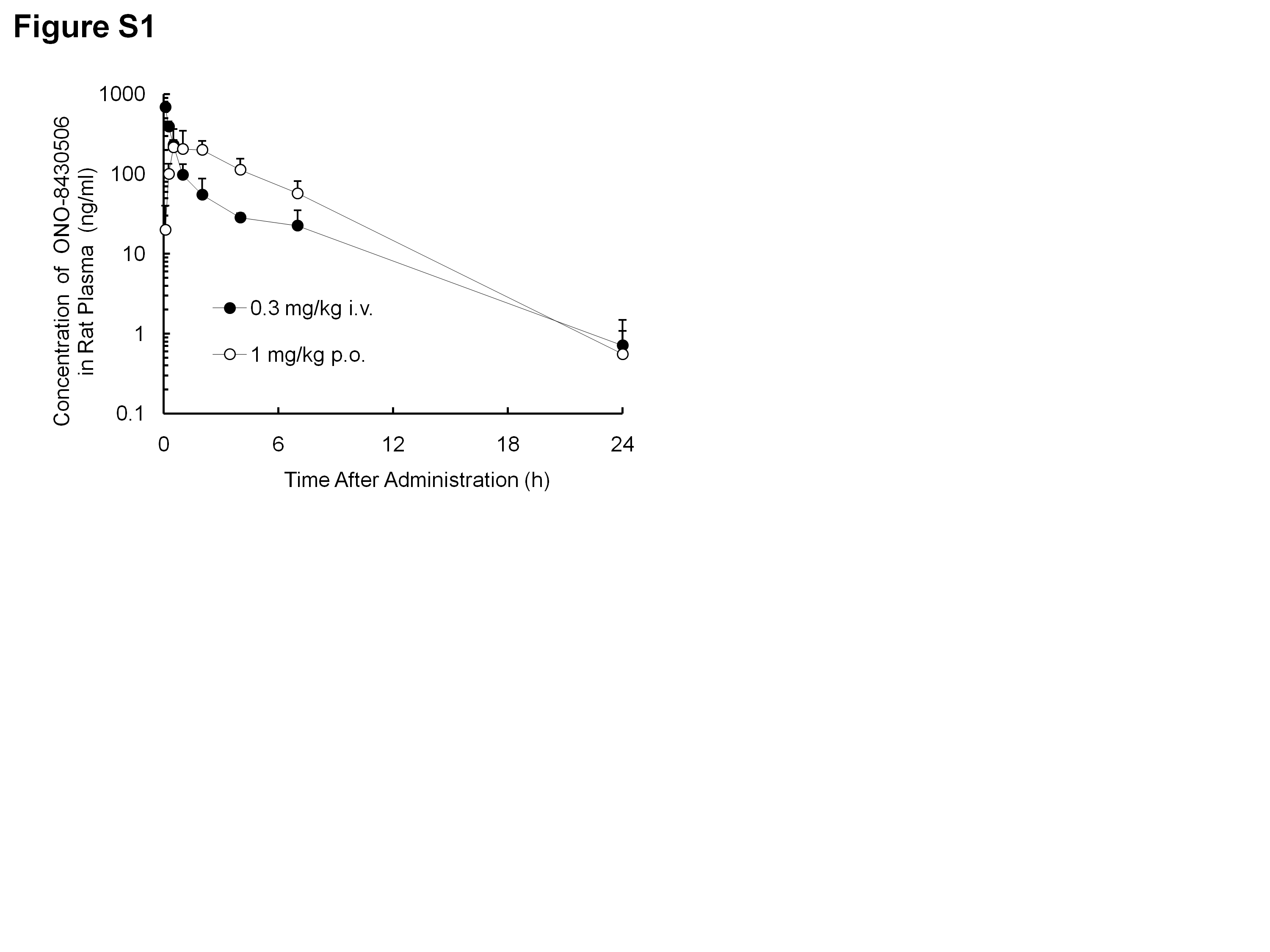

Supplement: Figure S1 — Pharmacokinetics in Rats. After intravenous (at 0.3 mg/kg) or oral (at 1 mg/kg) administration of a single dose of ONO-8430506 to rats under fasting conditions, blood was collected at various time points. The time course of changes in plasma concentration of unchanged compound is shown. The data are given as the mean ± S.D. for three rats in each dose group. (TIF) [file pone.0093230.s001.tif]

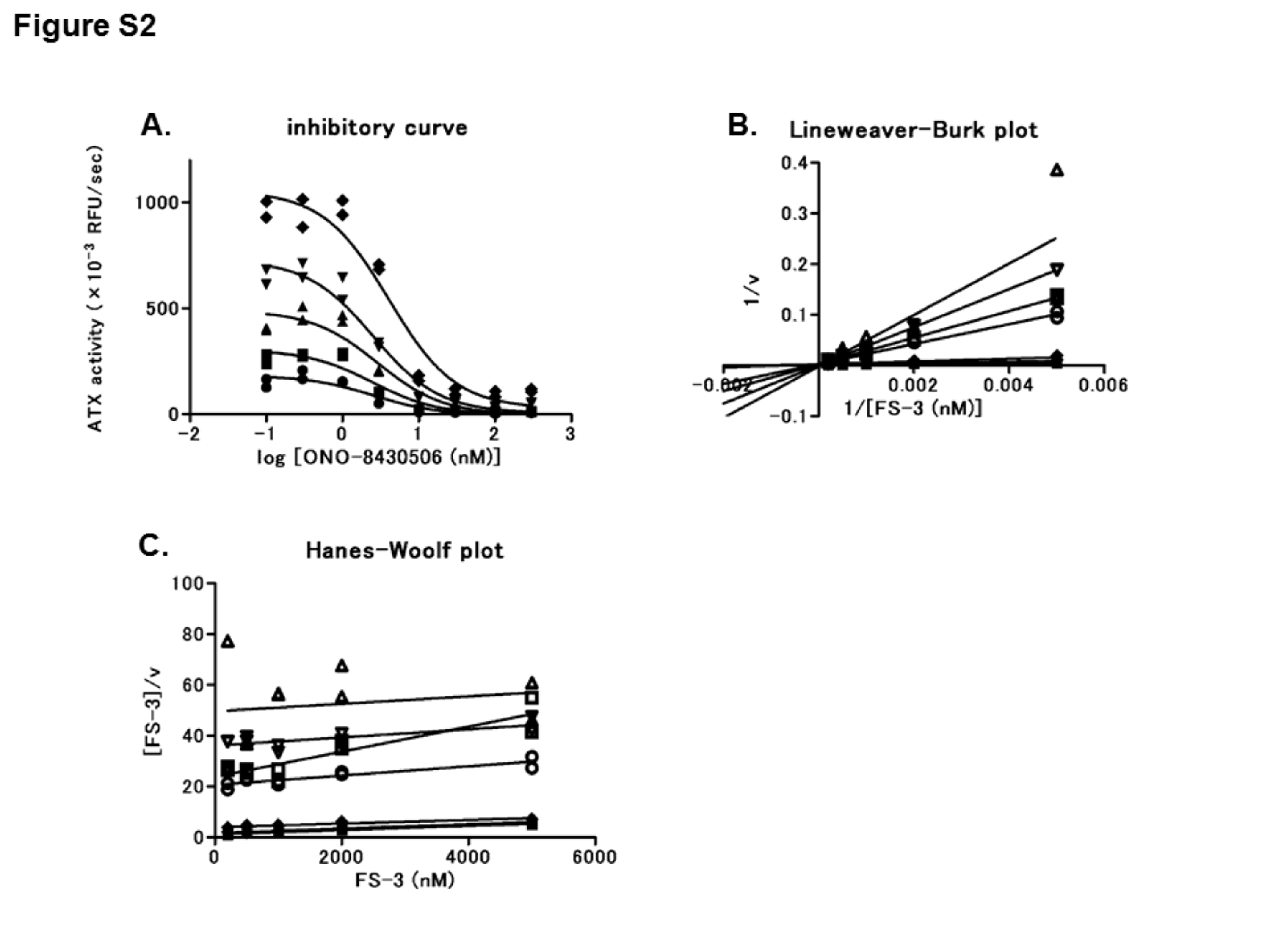

Supplement: Figure S2 — Enzyme Kinetics Analysis. (A) Inhibitory curves of recombinant human ATX/ENPP2 by ONO-8430506 for each concentrations of fluorescent substrate FS-3 (•: 200 nM, ▪: 500 nM, ▴: 1 µM, ▾: 2 µM, ♦: 5 µM). (B) Lineweaver-Burk plot analysis of ATX/ENPP2 inhibition by ONO-8430506 (•: no inhibitor, ▪: 100 pM, ▴: 300 pM, ▾: 1 nM, ♦: 3 nM, ○: 10 nM, □: 30 nM, ▵: 100 nM, ▿: 300 nM). (C) Hanes-Woolf plot analysis of ATX/ENPP2 inhibition by ONO-8430506 (•: no inhibitor, ▪: 100 pM, ▴: 300 pM, ▾: 1 nM, ♦: 3 nM, ○: 10 nM, □: 30 nM, ▵: 100 nM, ▿: 300 nM). ATX/ENPP2 inhibition type by ONO-8430506 is speculated as a competitive type, since Lineweaver-Burk plot crosses at a single point on Y-axis and Hanes-Woolf plot represents parallel lines. (TIF) [file pone.0093230.s002.tif]

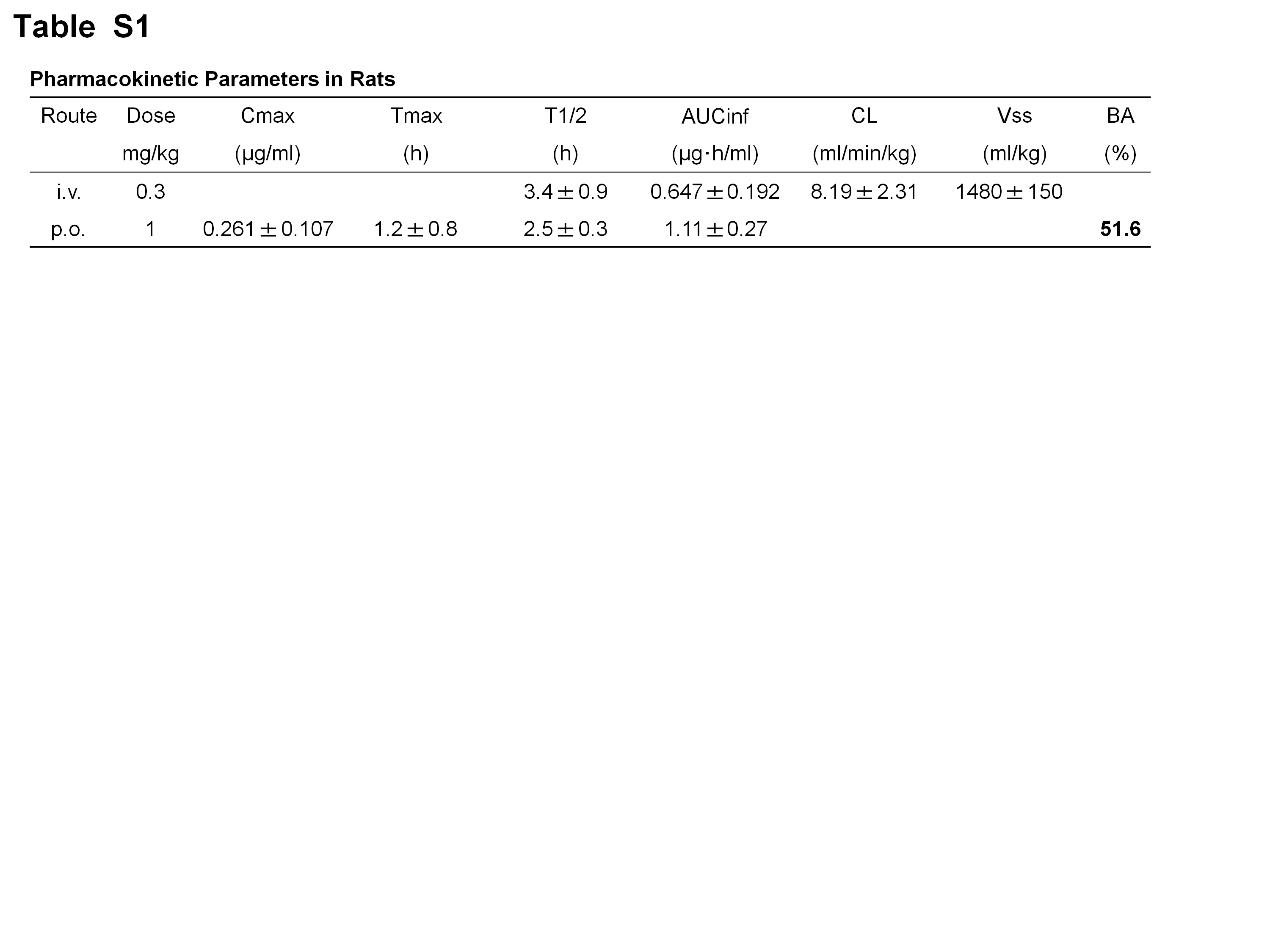

Supplement: Table S1 — Pharmacokinetics Parameter in Rats. From the time course of changes in plasma concentration of ONO-8430506 (shown in Figure S1), the pharmacokinetics parameters of the compound in rats were determined. (TIF) [file pone.0093230.s004.tif]
