# Supplementary material for: A Novel Highly Potent Autotaxin/ENPP2 Inhibitor Produces Prolonged Decreases in Plasma Lysophosphatidic Acid Formation In Vivo and Regulates Urethral Tension
Source: PLoS One. 2014 Apr 18;9(4):e93230. doi: 10.1371/journal.pone.0093230 (PMC3991570; doi:10.1371/journal.pone.0093230)
Supplement: File S1 — Contains the following: Method S1 and Method S2. (DOC) [file pone.0093230.s003.doc]

**Supporting Information**

**Methods**

**Method S1. Administration and blood sampling in rats for the PK study:**

A solution of ONO-8430506 for oral administration (hereafter, ‘p.o. solution’) was prepared by dissolving the compound in Wellsolve (Celeste Co., Ltd., Tokyo, Japan)/water (1:4) containing an equivalent molar amount of NaOH. Similarly, a solution of the compound for intravenous administration (hereafter, ‘i.v. solution’) was prepared by dissolving the compound in 30% 2-hydroxylpropyl-β-cyclodextrin (HP-β-CD) aqueous solution containing an equivalent molar amount of NaOH.

A single dose (1 mg/5 ml/kg) of ‘p.o. solution’ was given orally to 8-week-old male Crl:CD(SD) rats under fasting conditions overnight and heparinized blood was collected at various times from the jugular vein under awake, but constrained conditions. Similarly, a single dose of 0.3 mg/ml/kg of ‘i.v. solution’ was injected into the jugular vein of 8-week-old male Crl:CD(SD) rat under fasting conditions overnight and blood was collected in a similar manner. Blood samples were immediately cooled on ice and then centrifuged at 8,000*g* and 4˚C for 5 min. The plasma samples were stored at -20˚C and the plasma concentration of the compound was determined by LC-MS/MS to determine bioavailability.

**Method S2. Enzyme kinetics analysis:**

Recombinant human ATX/ENPP2 protein (2 μg/ml) and various concentrations of ONO-8430506 were mixed and incubated at 37˚C in 50 mM Tris-HCl buffer (pH 8.0) containing 1%DMSO, 140 mM NaCl, 5 mM KCl, 1 mM CaCl_2_, 1 mM MgCl_2_, and 1 mg/ml fatty acid free bovine serum albumin (Sigma-Aldrich Japan), in the presence of various concentration of fluorescent substrate FS-3. Fluorescence at 527 nm with excitation at 485 nm was measured in every 30 seconds for 1 hour. ATX activity was represented as relative fluorescence units (RFU) per second. Lineweaver-Burk plot and Hanes-Woolf plot were performed to speculate inhibition type of ATX/ENPP2 by ONO-8430506.

**Figure Legends**

**Figure S1. Pharmacokinetics in Rats**

After intravenous (at 0.3 mg/kg) or oral (at 1 mg/kg) administration of a single dose of ONO-8430506 to rats under fasting conditions, blood was collected at various time points. The time course of changes in plasma concentration of unchanged compound is shown. The data are given as the mean ± S.D. for three rats in each dose group.

**Table S1. Pharmacokinetics Parameter in Rats**

From the time course of changes in plasma concentration of ONO-8430506 (shown in Figure S1), the pharmacokinetics parameters of the compound in rats were determined.

**Figure S2. Enzyme Kinetics Analysis**

(A) Inhibitory curves of recombinant human ATX/ENPP2 by ONO-8430506 for each concentrations of fluorescent substrate FS-3 (●: 200 nM, ■: 500 nM, ▲: 1μM, ▼: 2μM, ♦: 5μM). (B) Lineweaver-Burk plot analysis of ATX/ENPP2 inhibition by ONO-8430506 (●: no inhibitor, ■: 100 pM, ▲: 300 pM, ▼: 1 nM, ♦: 3 nM, ○: 10 nM, □: 30 nM, ∆: 100 nM, ∇: 300 nM). (C) Hanes-Woolf plot analysis of ATX/ENPP2 inhibition by ONO-8430506 (●: no inhibitor, ■: 100 pM, ▲: 300 pM, ▼: 1 nM, ♦: 3 nM, ○: 10 nM, □: 30 nM, ∆: 100 nM, ∇: 300 nM). ATX/ENPP2 inhibition type by ONO-8430506 is speculated as a competitive type, since Lineweaver-Burk plot crosses at a single point on Y-axis and Hanes-Woolf plot represents parallel lines.
